# Understanding how and under what circumstances decision coaching works for people making healthcare decisions: a realist review

**DOI:** 10.1186/s12911-022-02007-0

**Published:** 2022-10-08

**Authors:** Junqiang Zhao, Janet Jull, Jeanette Finderup, Maureen Smith, Simone Maria Kienlin, Anne Christin Rahn, Sandra Dunn, Yumi Aoki, Leanne Brown, Gillian Harvey, Dawn Stacey

**Affiliations:** 1 School of Nursing, Faculty of Health Sciences, University of Ottawa, Ottawa, Canada; 2 School of Rehabilitation Therapy, Faculty of Health Sciences, Queen’s University, Kingston, Canada; 3 Department of Renal Medicine, Aarhus University Hospital, Aarhus, Denmark; 4 Department of Clinical Medicine, Aarhus University, Aarhus, Denmark; 5 Research Centre for Patient Involvement, Aarhus University & Central Region Denmark, Aarhus, Denmark; 6 Cochrane Consumer Network Executive, Ottawa, Canada; 7 Department of Health and Caring Sciences, Faculty of Health Sciences, UiT The Arctic University of Norway, Langnes, Norway; 8 Department of Medicine and Healthcare, The South-Eastern Norway Regional Health Authority, Hamar, Norway; 9 Nursing Research Unit, Institute of Social Medicine and Epidemiology, University of Lübeck, Lübeck, Germany; 10 BORN Ontario, Ottawa, Canada; 11 Children’s Hospital of Eastern Ontario Research Institute, Ottawa, Canada; 12 Ottawa Hospital Research Institute, Ottawa, Canada; 13 Psychiatric and Mental Health Nursing, Graduate School of Nursing Science, St. Luke’s International University, Tokyo, Japan; 14 School of Nursing, Queensland University of Technology, Brisban, Australia; 15 Caring Futures Institute, College of Nursing and Health Sciences, Flinders University, Adelaide, Australia

**Keywords:** Decision coaching, Shared decision making, Program theory, Realist review

## Abstract

**Background:**

Decision coaching is non-directive support delivered by a trained healthcare provider to help people prepare to actively participate in making healthcare decisions. This study aimed to understand how and under what circumstances decision coaching works for people making healthcare decisions.

**Methods:**

We followed the realist review methodology for this study. This study was built on a Cochrane systematic review of the effectiveness of decision coaching interventions for people facing healthcare decisions. It involved six iterative steps: (1) develop the initial program theory; (2) search for evidence; (3) select, appraise, and prioritize studies; (4) extract and organize data; (5) synthesize evidence; and (6) consult stakeholders and draw conclusions.

**Results:**

We developed an initial program theory based on decision coaching theories and stakeholder feedback. Of the 2594 citations screened, we prioritized 27 papers for synthesis based on their relevance rating. To refine the program theory, we identified 12 context-mechanism-outcome (CMO) configurations. Essential mechanisms for decision coaching to be initiated include decision coaches’, patients’, and clinicians’ commitments to patients’ involvement in decision making and decision coaches’ knowledge and skills (four CMOs). CMOs during decision coaching are related to the patient (i.e., willing to confide, perceiving their decisional needs are recognized, acquiring knowledge, feeling supported), and the patient-decision coach interaction (i.e., exchanging information, sharing a common understanding of patient’s values) (five CMOs). After decision coaching, the patient’s progress in making or implementing a values-based preferred decision can be facilitated by the decision coach’s advocacy for the patient, and the patient’s deliberation upon options (two CMOs). Leadership support enables decision coaches to have access to essential resources to fulfill their role (one CMOs).

**Discussion:**

In the refined program theory, decision coaching works when there is strong leadership support and commitment from decision coaches, clinicians, and patients. Decision coaches need to be capable in coaching, encourage patients’ participation, build a trusting relationship with patients, and act as a liaison between patients and clinicians to facilitate patients’ progress in making or implementing an informed values-based preferred option. More empirical studies, especially qualitative and process evaluation studies, are needed to further refine the program theory.

**Supplementary Information:**

The online version contains supplementary material available at 10.1186/s12911-022-02007-0.

## Background

Decision coaching helps people prepare to actively participate in making health decisions [1–4]. It is relational and non-directive support delivered by a trained healthcare provider using face-to-face, telephone, or other communication media (i.e., not automated electronically) [1, 4]. The goal is to improve the decision making process and to ultimately help people to achieve informed and values-congruent decisions. Decision coaches: (a) identify the persons’ decision making needs; (b) help them understand evidence-based information on options (including status quo), benefits, and harms; (c) clarify their values for outcomes of options (what matters to them); and (d) encourage them to communicate their preferences to others (e.g., family, clinicians).

Theoretical frameworks and models have been used to guide decision coaching interventions, among which four were commonly used [2, 3]. The Ottawa Decision Support Framework (ODSF) proposes that a decision support intervention such as decision coaching used to address patients’ decisional needs will improve the quality of decision making process and the decision, which may favorably affect the implementation of the chosen option and use of health services [5]. The Framework for Decision Coach-Mediated Shared Decision Making [8] positions the decision coaching intervention within a triadic decision making relationship between clinicians, patients, and decision coaches. In the Situation–Choices–Objectives–People–Evaluation–Decisions (SCOPED) framework, a non-directive trained facilitator elicits the patient’s questions without providing information or advice, and produces a written question list organized according to the SCOPED topics for the patient to use in consultations with their healthcare providers [7, 9, 10]. The International Patient Decision Aids Standards checklist has a subset of two criteria for coaching in deliberation and communication used alongside a patient decision aid: (a) the option of working with a trained coach to help patients consider the options; and (b) the use of the trained coach to help patients prepare to talk about the decision with a practitioner [6]. Other theoretical frameworks and models, such as the Six-step Shared Decision Making Model, have also been reported to guide the design of decision coaching interventions [11–13]. While these frameworks provide theoretical support for developing coaching interventions to promote patient progress in decision making, they do not consider the unique working mechanisms of decision coaching and its differences from other evidence-based patient decision support interventions (e.g., patient decision aids). Thus, these frameworks offer a limited explanation of the mechanisms by which decision coaching interventions work.

There is some evidence on the effectiveness of decision coaching, but most studies evaluated it as an intervention used alongside evidence-based written information [3, 14]. A recent Cochrane systematic review identified 28 randomized controlled trials on decision coaching. This review reported that when used alone or with evidence-based information to help people prepare to make healthcare decisions, decision coaching may improve participants’ knowledge without any significant adverse events (e.g., decision regret, anxiety) [2]. There were variable effects on other outcomes, such as preparation for decision making and decision self-confidence [2].

While it has been possible to identify studies to test the effectiveness of decision coaching, we do not yet know the circumstances (that is, the context) under which decision coaching interventions can contribute to people's progress in making healthcare decisions. More importantly, we do not know how or through what mechanisms decision coaching interventions may (or may not) make change happens. By understanding the contexts and mechanisms for decision coaching interventions to work, we can better rationalize the use of human resources on such interventions and develop theory-informed interventions that can be tailored within different contexts. The purpose of this study was to understand how and under what circumstances decision coaching works for people making healthcare decisions.

### Research questions

1. What are the possible mechanisms by which decision coaching results in intended outcomes?
2. What are the contextual factors that activate the decision coaching mechanisms to produce intended outcomes?

## Methods

We used a realist review methodology to answer the research questions [15, 16]. Realist review is a theory-driven evidence synthesis methodology designed to unpack the “black box” of complex social interventions [17] and to provide explanations as to “what works for whom, in what circumstances, in what respects and how”, operationalized as context-mechanism-outcome (CMO) configurations (i.e., an outcome of interest [O] is generated by relevant mechanisms [M] being activated in specific contexts [C]) [15, 18]. A realist review methodology fits with the study purpose because it helps to uncover the mechanisms through which decision coaching works, the contextual factors that activate the mechanisms, and subsequent outcomes. This realist review was built on a Cochrane systematic review which assessed the effects of decision coaching in people facing healthcare decisions [1, 2]. The review findings were presented based on the Realist and Meta-Review Evidence Synthesis reporting guideline [19].

**Fig. 1:**
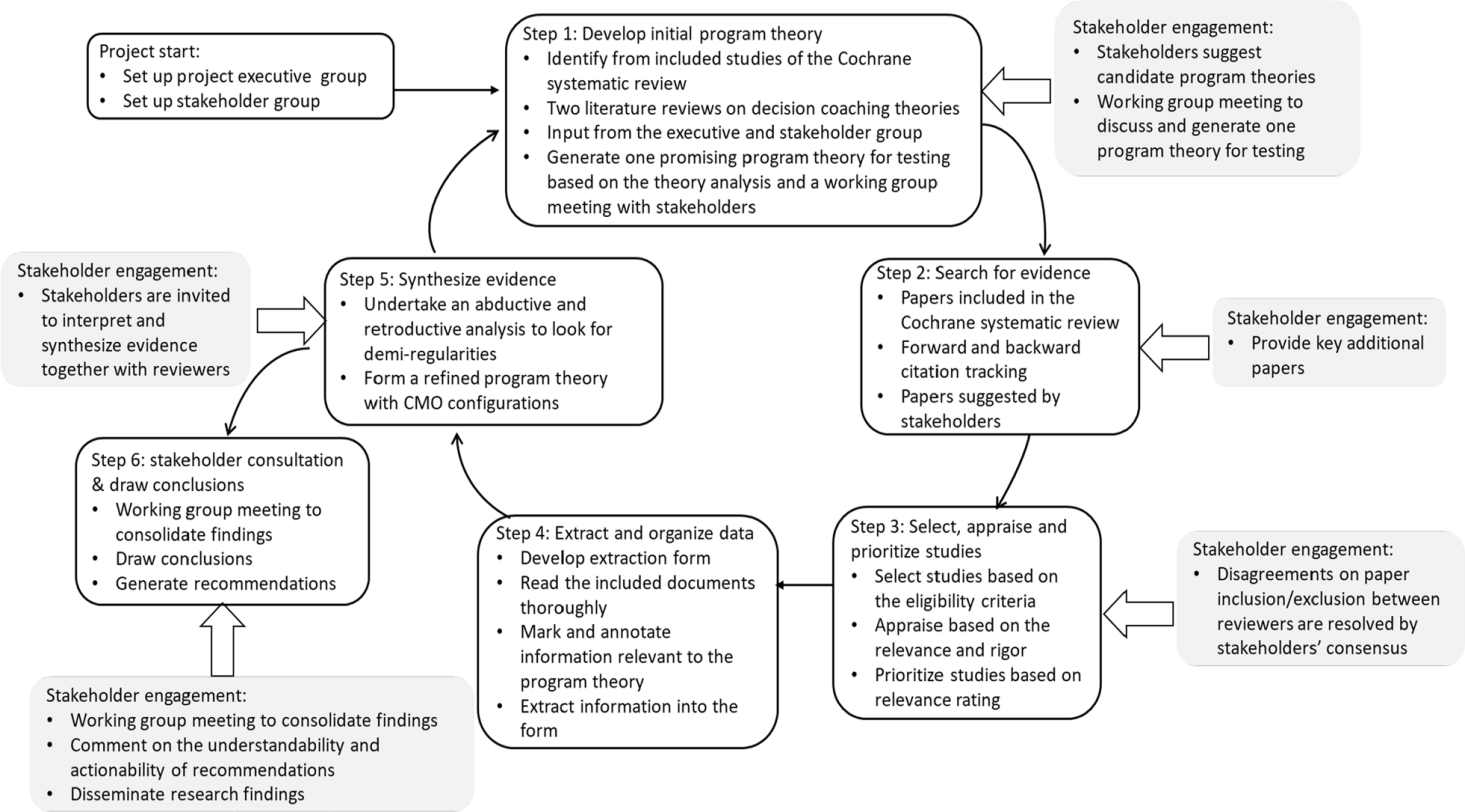
Flow diagram of the project

For our realist review, we set up a project executive group (JZ, JJ, DS) and a stakeholder group (JF, SK, AR, SD, YA, LB, GH,MS) from six countries (i.e., Australia, Canada, China, Denmark, Germany, Japan, Norway) with a healthcare consumer (MS), all of whom have variable experiences in decision coaching (Additional file 1: Appendix A). Our team members included healthcare professionals who provided decision coaching, educators who offered training in decision coaching, researchers who were involved in conducting studies to evaluate decision coaching and other decision support interventions, and one healthcare consumer who experienced decision coaching as an intervention. The stakeholder group members were engaged throughout the realist review process as content experts. The review involved six iterative steps [15, 16] as follows (see Fig. 1).

### Step 1: develop the initial program theory

The project executive group drafted an initial program theory that explained how decision coaching was thought to work and to generate the outcomes of interest from three sources: theories reported in the 28 included studies of the Cochrane systematic review [2]; theories identified in two reviews of decision coaching theories [3, 14]; and theories provided by the executive and stakeholder group members. We screened those theories based on the criterion: whether the theory provided an explanatory focus to help uncover the mechanism(s) of how decision coaching works, rather than a descriptive account of the decision coaching process. Next, we identified context, mechanism, and/or outcome relevant concepts or propositions from included theories and generated one promising program theory for a stakeholder group meeting. We developed an initial program theory after integrating feedback from a one-hour stakeholder group meeting (see Additional file 1: Appendix B). This initial program theory was used for testing and refinement through a review of literature at later stages.

### Step 2: search for evidence

Different from systematic reviews which require a comprehensive and exhaustive search of literature, realist reviews “seek to sample literature and attain modest forms of theoretical generalisability from evidence” (page 149) [20] through a purposive approach and do not intend to achieve comprehensiveness [21]. Therefore, rather than restarting a comprehensive literature search, the project executive group retrieved evidence from four existing literature sources that were closely related to this topic: (a) 28 randomized controlled trials examining the effectiveness of decision coaching interventions included in the Cochrane systematic review [2]; (b) for these 28 studies, a forward citation search was conducted using Google Scholar and a backward citation search was conducted by screening the reference lists; (c) 36 studies excluded at the full-text screening stage of the Cochrane systematic review due to having decision coaching in both groups (n = 25), using non-randomized-controlled-trial designs (n = 9), and coaching by groups (n = 2); (d) papers identified by stakeholder group members that had been used to guide their decision coaching research, practice or teaching.

### Step 3: select, appraise, and prioritize studies

The research team developed eligibility criteria for the realist review based on the criteria used in the Cochrane review [2], but were intentionally more inclusive of study designs. The inclusion criteria for selecting literature were (a) population: adults and children preparing to make a healthcare decision regarding treatment, screening, or diagnosis for themselves or a family member; (b) intervention: non-directive support by a healthcare provider who helps to prepare the patient to make a health decision; (c) empirical studies and their study protocols (protocols without implementation and evaluation data were not considered); (d) papers in any language. The exclusion criteria were: (a) population: simulated patients and those in which healthcare providers are making the decision with, or on behalf of, the patient; are recommending a specific treatment; are not described as having direct interests in providing decision coaching; (b) intervention: decisions about lifestyle choices, participation in research, hypothetical situations, genetic testing, clinical problems where there is only one reasonable option, or advanced care planning; papers that describe automated support; (c) study designs: dissertations, conference proceedings, editorials, commentaries, and book chapters.

Two reviewers (JZ, DS) independently screened titles and abstracts based on the eligibility criteria using Covidence (https://www.covidence.org), and conflicts were resolved by a third reviewer (JJ). Full texts of these potentially eligible studies were retrieved and independently assessed for eligibility by two reviewers (JZ, JJ). Disagreements were discussed and resolved by consensus or presented to the stakeholder group to determine eligibility. A Preferred Reporting Items for Systematic Reviews and Meta-Analyses (PRISMA) flow diagram was completed to record the number of papers identified, screened, and included for full-text review [22].

Two reviewers (JZ, JJ) appraised the quality of included studies independently from two dimensions: relevance and rigour [23], and discrepancies were resolved through discussion or a third-party adjudication (DS). For relevance, we developed a relevance rating checklist based on the propositions of the initial program theory with four items: level of relevance with mechanisms, contexts, outcomes, and the overall contribution to the understanding of decision coaching. Each item contained four statements to represent four different levels of strength of relevance, similar to a four-point Likert scale (i.e., level 3: high relevance; level 0: no relevance) (see Additional file 1: Appendix C). We grouped highly relevant papers (i.e., three points for the overall level of relevance score) as a priority for data extraction in that they provided richer information to understand decision coaching mechanisms. For rigour assessment of randomized controlled trials, we used the Cochrane risk of bias appraisal tool [24]; for other studies (such as non-randomized controlled trials), we used the Mixed Methods Appraisal Tool [25]. Papers were not excluded based on the methodological qualities, but rather, this assessment provided insight into the rigour of included studies.

### Step 4: extract and organize data

The project executive group developed a customized data extraction form based on the propositions of the initial program theory, which was adjusted based on a pilot extraction of two included studies. The form was used to not only obtain basic characteristics of included studies, but more importantly, capture information on the context, proposed/described mechanisms of action, and outcomes (see Additional file 1: Appendix D). Starting with highly relevant papers, all the team members participated in data extraction with JZ and JJ checking the accuracy of extraction.

### Step 5: synthesize evidence

We synthesized the evidence through a two-phase process: within-study analysis and cross-study analysis. In the within-study analysis phase, two reviewers (JZ, JJ) immersed themselves into the texts to become familiarized and independently identified relationship statements that reflected the potential connection between any two of the contexts, mechanisms, and outcomes [26, 27]. The two reviewers met, comparing and integrating the relationship statements to generate a set of refined relationship statements for each paper or cluster of papers (e.g., protocol and the trial), and extracted the corresponding contexts, mechanisms, and outcomes. We classified the relationship statements into three decision coaching stages (i.e., before, during, after decision coaching) involving three key roles (i.e., patient, decision coach, clinician) and formulated initial CMO statements based on the recurrent patterns within those relationship statements. In the cross-study analysis phase, we (JZ, DS, JJ, JF) held a two-hour working group meeting to discuss the initial CMO statements, and generated a set of refined CMO statements and a refined program theory.

Three main strategies were used to assist the synthesis: juxtaposition, reconciliation, and adjudication [28–30]. When some studies revealed underlying mechanisms, some concentrated on outcomes, and others described context in-depth, we juxtaposed data sources to build theoretical understanding. When contradictory findings were found between studies, we reconciled data sources to identify contextual and implementation differences to explain opposing outcomes, or adjudicated them based on methodological strengths or weaknesses.

During the synthesis process, abductive and retroductive reasoning approaches were used to analyze data and look for recurring patterns [31, 32]. The abduction was used primarily in the within-study analysis phase where two reviewers paid special attention to the texts on contexts or mechanisms that were not manifested in the initial program theory and identified patterns. The retroduction was used in the cross-study analysis phase (and step 6) where the reviewers and stakeholders worked together to uncover the hidden mechanisms through the logical analysis of the included papers and the use of personal experiences and prior knowledge.

### Step 6: consult stakeholders and draw conclusions

The project executive group sent the synthesis findings to all stakeholders with two probing questions for a planned group discussion: based on your experience in decision coaching, do these CMOs and the refined program theory make sense to you? Where can they be optimized to make better sense? We held a one and half-hour working group meeting with all stakeholders to fine-tune the CMOs and program theory. Using a consensus-based approach, this process primarily focused on the optimization of wording and phrasing of CMO statements, and the refinement of contextual factors that activate the generative mechanisms without a major change of the mechanisms that were identified from the literature review.

## Results

### Basic characteristics of synthesized papers

**Fig. 2:**
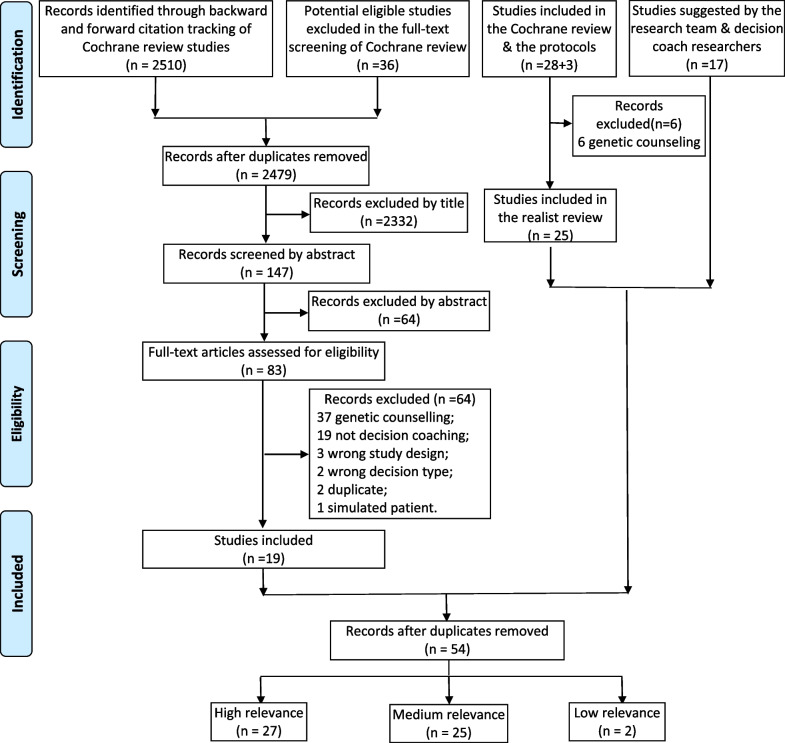
PRISMA diagram

Of the 2594 citations retrieved from four different literature sources, a total of 54 papers were included, among which 27 papers from 22 distinct studies were rated as highly relevant and used for synthesis [11–13, 33–56] (see Fig. 2). Various types of study designs were used in the 27 papers including randomized controlled trials (n = 14), pre-post studies (n = 4), qualitative studies (n = 3), study protocols (n = 3), mixed-method pilot study (n = 1), process evaluation (n = 1), non-randomized comparative study (n = 1). The prioritized studies were conducted in the USA (n = 8), Canada (n = 4), Australia (n = 3), Germany (n = 2), Scotland (n = 2), UK (n = 1), Netherlands (n = 1), and multinational (n = 1) (see Table 1).

**Table 1:** Basic characteristics of the synthesized papers (n = 27)

First author, year	Country	Study design	Guiding theory	Setting	Providers (N)	Consumers (N)	Decision type	Intervention
Berger-Höger, 2015 [12]	Germany	Study protocol for a cluster RCT	Six-step Shared Decision Making Model	Certified breast care centers	Specialized breast care and oncology nurses (n/r)	Patients with ductal carcinoma in situ (N=192 planned)	Treatment of ductal carcinoma in situ	Patient decision aid, at least one decision coaching session and a final shared decision making physician encounter
Berger-Höger, 2017 [13]	Germany	Intervention development & mixed method pilot	Same with above	Two breast care centres	Specialized breast care and oncology nurses (N=4)	Patients with ductal carcinoma in situ (N=7)	Same with above	Same with above
Berger-Hoger, 2019 [11]	Germany	Cluster RCT	Same with above	16 certified breast care centers	Specialized breast care and oncology nurses (N=31)	Patients with ductal carcinoma in situ (IG (N=37) versus CG (N=30))	Same with above	Same with above
Brown, 2016 [33]	Australia	Study protocol for an RCT	ODSF	Four public health renal departments in Queensland	Trained renal nurse (n/r)	Older patients with advanced kidney disease (N=122 planned)	Dialysis or conservative kidney management	A workbook, audio recording, personal worksheet and consultation with a trained renal nurse
Brown, 2019 [34]	Australia	Pragmatic RCT	ODSF	Four public health renal departments in Queensland	Registered Nurse (N=1)	Older patients with advanced kidney disease (IG (N=19) versus CG (N=22))	Same with above	Same with above
Causarano, 2015 [35]	Canada	Pilot RCT	ODSF	A tertiary cancer center in Toronto, Canada	A plastic surgeon, a nurse specialist, a social worker, and two peer support patients (n/r)	Patients undergone mastectomy (IG (N=21) versus CG (N=20))	Postmastectomy breast reconstruction	Pre-consultation educational group intervention (treatment options; pre- and postoperative care; values clarification; peer experience sharing)
Davison, 1997 [36]	Canada	RCT	The Empowerment Model by Conger and Kanungo	Community urology clinic	Physicians (n/r)	Men with prostate cancer (N=60)	Prostate cancer treatment	A written information package and medical consultation
Feenstra, 2015 [37]	Canada	Pre-/post-test	ODSF, OFDG	An ambulatory diabetes clinic in a tertiary children’s hospital	Diabetes social workers (N=2)	Families with the children suffer from type 1 diabetes (N=7)	Insulin delivery options	Decision coaching
Hacking, 2013 [38]	Scotland	RCT	SCOPED	One hospital diagnostic clinic	Research assistants (N=2)	Early-stage prostate cancer patients (IG (N=63) versus CG (N=60))	Treatment decisions for early-stage prostate cancer	Decision navigation
Holt, 2009 [39]	USA	Cluster RCT	Social Cognitive Theory, Health Belief Model	Two area Baptist churches	One trained community health advisor from each church (n/r)	African American men who had not had prostate cancer (IG (N=31) versus CG (N=18))	Prostate cancer screening	An educational session and distributed educational print materials
Ilic, 2018 [40]	Australia	Qualitative study	n/r	n/r	Practice nurses (N=12) & general practitioners (N=16)	Men with prostate cancer (N=19)	Prostate cancer screening	n/r
Johnson, 2010 [41]	Nicaragua, Mexico & Indonesia	Pre/post study	Client-centered counseling principles	49 government health facilities in Nicaragua; 9 government health facilities in Mexico City; 6 public health clinics in Indonesia	In Nicaragua: Healthcare providers (N=59); In Mexico: doctors (N=9), nurses (N=2), social workers (N=2); In Indonesia: midwives (N=12)	Family Planning Clients (n/r)	Family Planning method	A 2- to 4-day training workshop for providers to introduce the Tool and then use of the Tool in routine work for a time (4 months in Nicaragua, 1 month in Mexico and Indonesia)
Jull, 2015 [42]	Canada	Qualitative study	ODSF	Minwaashin Lodge	n/r	Indigenous women (N=19)	Neutral decision with health impact	n/r
Kearing, 2016 [43]	USA	RCT	ODSF	Orthopaedic spine clinic	Nurse, genetic counselor, social workers (n/r)	Patients with lumbar spinal stenosis (IG (N=98) versus CG (N=101))	Treatment of spinal stenosis	Video decision aid plus health coaching
Lawson, 2020 [44]	Canada	Pre/post study	ODSF, OFDG	Pediatric diabetes clinic in a tertiary care centre	Social workers (N=2)	Youth (N=45) and parents (N=66)	Insulin delivery options	Decision coaching
Lenzen, 2018 [45]	Netherlands	Process evaluation	A framework for shared decision making about goals and actions, a 4-circles tool	Regional family medicine organization	Practice nurses (N=15)	Patients (N=10)	n/r	n/r
Lepore, 2012 [46]	USA	RCT	ODSF	A large healthcare workers’ union	Graduate students with training in public health and health education (n/r)	Immigrant Black Men (IG (N=244) versus CG (N=246))	Prostate cancer screening	Educational pamphlet and a maximum of two tailored telephone education
Lowenstein, 2020 [47]	USA	Pre/post study	ODSF	3 radiology clinics	Advanced practice providers (N=4), research nurse (N=1), and radiologist (N=1)	Patients (N=81)	Lung cancer screening	Patient decision aid and decision coaching
McBride, 2016 [48]	UK	RCT	SCOPED	1 diabetes foot clinic	Health psychologists (N=5)	Patients with a diabetic foot ulcer (IG (N=30) versus CG (N=26))	Treatment of diabetic foot ulcer	Decision Navigation
Mishel, 2009 [49]	USA	RCT	Uncertainty of Illness Theory	Prostate cancer treatment centres	Nurse (N=1)	Men (IG (N=93) versus CG (N=74))	Prostate cancer treatment	A booklet, a DVD demonstrating communication skills, 4 coaching calls
Rahn, 2015 [51]	Germany	Study protocol for a cluster RCT	Six-step Shared Decision Making model	Neurological outpatient clinics throughout Germany	Nurses specialising in multiple sclerosis (n/r)	Patients older than 18 years with possible multiple sclerosis (N=300 planned)	Immunotherapy decision	Decision coaching
Rahn, 2018 [50]	Germany	Feasibility testing, pilot RCT, & mixed methods process evaluation	Same with above	Two pilot multiple sclerosis centres in Germany	Nurses specialising in multiple sclerosis (N=4)	People with possible multiple sclerosis (IG (N=38) versus CG (N=35))	Immunotherapy decision	Decision coaching
Rothert, 1997 [52]	USA	RCT	A conceptual framework for decision support	A midwestern university community	Physician (N=1), nurses (N=3) psychologists (N=2) and health services researcher (N=1)	Women ((IG (N=83) versus CG1 (N=87)) versus CG2 (N=78))	Management of menopausal symptoms and hormone replacement therapy	Brochure, structured lecture and discussion, and tailored decision support intervention
Shepherd, 2019 [53]	Scotland	RCT	SCOPED	One clinic in a cancer centre	Research psychologists (N=2)	Colorectal cancer patients (IG (N=68) versus CG (N=69))	Treatment of colorectal cancer	Consultation planning, summary and audio recording
Sheridan, 2012 [54]	USA	RCT	n/r	4 primary care practices	Health counselor (N=1)	Men (IG (N=60) versus CG (N=70))	Prostrate cancer screening	video-based decision aid and researcher-led coaching session
Simmons, 2017 [55]	Australia	Non- randomized comparative study	ODSF and IPDAS criteria	Youth mental health service in New South Wales Australia	Peer support workers (n/r)	Young people (IG (N=149) versus CG (N=80))	Mental health	Decision support using an online tool
Thom, 2016 [56]	USA	Qualitative study	n/r	6 urban public health primary care clinics	Medical assistants or other allied nonlicensed health workers (N=17)	Low-income patients with chronic conditions (N=30 for focus group, N=42 for individual interview)	n/r	n/r

RCT = randomized controlled trial; ODSF = Ottawa Decision Support Framework; OFDG = Ottawa Family Decision Guide; SCOPED = Situation, Choices, Objectives, People, Evaluation, and Decisions; IPDAS = International Patient Decision Aids Standards; IG = Intervention Group; CG = Control Group; n/r = not reported

The most commonly used theories to guide decision coaching interventions were the ODSF (n = 8/22), followed by the SCOPED framework (n = 3/22) and the Six-step Shared Decision Making Model (n = 2/22). Included studies addressed a range of healthcare decisions, such as the treatment of prostate cancer, breast cancer, and spinal stenosis, that occurred in study settings, such as urology clinics, cancer care centres, and orthopedic spine clinics. Decision coaching was provided by nurses, physicians, social workers, psychologists, and a mix of healthcare professionals.

### CMO configurations for decision coaching

We developed 12 CMO statements based on the realist review of literature and reflecting discussions among team members (see Table 2). To avoid redundancy, we used the term “patients” within these CMOs to represent persons, people, and health consumers who face a healthcare decision and participant in decision coaching.

**Table 2:** CMO configurations

	Context	Mechanisms	Outcomes	Supporting evidence	Quotes
Before decision coaching					
CMO1: Healthcare providers commit to decision coaching	Healthcare providers’ attitudes towards supporting patients in decision making and beliefs in patients’ roles, motivations, and abilities in decision making	Role commitment	Healthcare providers implement decision coaching, or collaborate with decision coaches to support their patients, and advocate for decision coaching to patients	[12, 11, 13, 40, 45, 56]	*“We found that the practice nurses’ attitudes influenced the implementation [decision coaching]. The nurses were frequently doubtful about their patients’ abilities and motivation to set goals and formulate action plans.”* [45]
CMO2: Healthcare providers develop knowledge and skills in providing decision coaching	Healthcare providers are trained and practice decision coaching	Knowledge and skills	Healthcare providers implement decision coaching	[12, 11, 13, 40, 45, 56, 33, 34, 37, 38, 39, 41, 42, 44, 46, 47, 48, 49, 50, 51, 53, 54]	*“Overall, decision coaches were satisfied with the training, found it informative and felt well prepared for the decision coaching, but the nurses suggested reducing the length of the training.”[*50*]*
CMO3: Patients are open to engage with a decision coach	Patients’ understanding of or experience with decision coaching	Valuing and willing to engage in decision coaching	Patients have decision needs addressed, progress in decision making, and increase the likelihood of future participation	[40, 34, 38, 50]	*“Some men were familiar with the concept [decision coaching], as they had experience dealing with a PN [practice nurse] for other health issues, such as diabetes checks. Men believed that it would provide them with an opportunity to speak to a health professional for an extended period of time, prior to consulting their GP [general practitioner].”*[40]
CMO4: Three roles (i.e., decision coach, patient, clinician) share a common goal of the patient being involved in decision making	The three roles are committed to patients' involvement in decision making	Sharing a common goal	They work collaboratively as partners in decision making process	[13, 40, 45, 48, 36]	*“Findings from our study… highlighting the relationship that needs to be built between GP [general practitioner], PN [practice nurse] and patient with respect to the decision-making process” *[40]
During decision coaching					
CMO5: Patients confide in the decision coach as a trusting relationship is built	There is a trusting relationship between a decision coach and a patient	Accepting coaching and willing to confide in the decision coach	Patients progress in decision making	[13, 56, 37, 42, 50, 51]	*“A positive relationship based on trust was seen as central to the coach’s ability to support the patient. A trusting relationship enabled patients to be honest, ask questions, and express doubts or disagreements, which allowed the health coach to be more effective” [*56*]*
CMO6: Patients perceive their decisional needs are recognized by the decision coach	A tailored approach to decision coaching, rather than a standardized protocol-based approach	Perceiving their needs are recognized	Patients progress in decision making	[45, 33, 41, 42, 44, 46, 47, 48]	*“The approach* [supporting nurses to coach patients in shared decision making about goals and actions] *is based on the following key principles: 1) Shared decision making is central to goal setting and action planning, asking for: a. a holistic exploration of the patient’s perspective;* *b. tailored coaching of the patient” [*45*]*
CMO7: Patients acquire knowledge for making the decision	A decision coach discusses evidence-based information on options, benefits, and harms with a patient	Exchanging information with the coach, asking questions, and acquiring knowledge	The patient improves understanding of information, and the quality of decision-making process will be improved	[12, 11, 13, 40, 45, 56, 33, 34, 37, 38, 39, 41, 42, 44, 46, 47, 48, 49, 50, 51, 53, 54, 36, 35, 43, 52, 55]	*“To make informed decisions, patients need to have a basic understanding of their condition, their options, and the consequences of each option. Health coaches provided education using patient-centered techniques that included determining patients’ goals and readiness for change and checking for patients’ understanding. Education was seen as playing a critical role in coaching support” [*56*]*
CMO8: The patient and decision coach reach a common understanding of patient’s values	A decision coach works with a patient to clarify the patient’s values for outcomes of options	Reaching a common understanding of what matters most to the patient	The patient become clearer on their values-based preferred option	[12, 11, 13, 40, 45, 56, 33, 34, 37, 39, 41, 42, 44, 46, 47, 48, 50, 51, 53, 54, 55]	*“The child’s preferences and values were elicited [by the decision coach] prior to their parent(s) to encourage child involvement and to help avoid biasing the child’s responses. The decision coach facilitated discussions between parent and child regarding agreement/ disagreement on values for benefits and harms of the options”[*44*]*
CMO9: Patients feel supported when family and significant others participate in decision coaching	Patients who prefer to work with supportive others, invite family members or significant others to participate in decision coaching together with the patient	Feeling supported	Patient selects a preferred option	[49, 36]	*“Several possible explanations exist as to why men who received the intervention were willing to assume more ownership for their treatment decision…Another explanation is that wives and/or other family members provided the men with the extra support and confidence they needed to assume a more active role in treatment decision making” [*36*]*
After decision coaching					
CMO10: Patients process information and deliberate on options	Patients, especially those with complex decisional needs*,* may need time to process information after a tailored decision coaching session(s)	Deliberating on options	Patients progress in decision making to reach a preferred option	[34, 38, 48, 53, 43, 52]	*“Additional exposure to OPTIONS and decision support helped to reduce uncertainty at T2. This effect illustrates that over time OPTIONS can support patient autonomy and reduce uncertainty, in a shared decision‐making model” [*34*]*
CMO11: Decision coaches share patients’ personal circumstances and advocate for their values for preferred options	A decision coach understands the impact of patient’s health condition on their quality of life and their values for outcomes of options	Playing a bridging role between the patient and clinician by sharing patient’s personal circumstances and advocating for patient's values for preferred option	Patient-clinician communication will be improved	[56, 53]	*“Bridging Between the Patient and Clinician–-The last theme refers to coaches working in conjunction with the clinicians to support patient decisions. It included improving patient understanding and communication with the clinician, helping the patient identify and ask questions of the clinician, supporting the patient between visits, and reducing the patient’s fear and anxiety around office visits” [*56*]*
Organization context for decision coaching					
CMO12: Decision coaches are supported by the leadership team with access to resources to fulfill their role	The organizational leadership team is committed to support patients' involvement in decision making	Having access to resources	The implementation and optimization of decision coaching	[11, 40, 45, 56, 34, 37, 41, 44, 50]	*“Participants in our study had some reservations about the feasibility of implementing a decision coaching intervention in primary practice, citing potentially increase in staff numbers, upskilling of health professionals’ knowledge, resources and cost as primary barriers to its uptake”[*40*] *

### CMO1: Healthcare providers commit to decision coaching

*When healthcare providers hold positive attitudes towards supporting patients in decision making and believe in patients’ roles, motivations, and abilities in decision making (C), they will commit to the decision coaching role (M), and as a result, implement and advocate for decision coaching, or collaborate with decision coaches to support their patients (O).* Six papers supported the theoretical statement in CMO1 [11–13, 40, 45, 56]. In their qualitative study, Ilic and colleagues found that practice nurses supported decision coaching to men on prostate cancer screening because they thought it “aligned with their current roles in primary practice” (page 875) [40]. In Berger-Höger and colleagues’ pilot study [13] and randomized controlled trial [11], they indicated that healthcare providers’ (including the decision coach and clinician) attitudes on supporting patients in decision making largely impacted the implementation of decision coaching for women with ductal carcinoma. Thom and colleagues also identified through a qualitative study that the personal commitment by decision coaches to help the patient was one key mechanism for supporting patients’ decision making [56].

### CMO2: Healthcare providers develop knowledge and skills in providing decision coaching

*When healthcare providers are trained and practice decision coaching (C), they will develop knowledge and skills (M), and as a result, implement decision coaching (O).* Twenty-two papers explicitly described the training for decision coaches before providing decision coaching [11–13, 33, 34, 37–42, 44–51, 53, 54, 56]. As Johnson and colleagues stated, “counseling [i.e., coaching] is a complex skill to master, needing training and practice” (page 360) [41]*.* Healthcare providers are likely to improve their knowledge and skills to implement decision coaching through training and practice. Rahn and colleagues further concluded in a pilot randomized controlled trial that healthcare providers trained in decision coaching had the potential to increase informed choice and participation as well as the effectiveness of patient-clinician consultation [50].

### CMO3: Patients are open to engage with a decision coach

*When patients have a basic understanding of or positive experience with decision coaching (C), they will value the benefit and engage with a decision coach (M), and as a result, have their decisional needs addressed, progress in decision making and increase the likelihood of future participation (O).* Four papers contributed to the theoretical statement in CMO3 [34, 38, 40, 50]. Three trials [34, 38, 50] explicitly reported the high rate of patients declining to meet with the decision coach for different reasons, such as “unwilling to be involved in decision making” (page 3037) [34], “too much effort to have further appointments” (page 30) [50], and “having already made a treatment decision” (page 1019) [38]. One qualitative study suggested that when patients had a positive experience with decision coaching, they were very likely to participate again [40]. Therefore, we propose that patients’ understanding and valuing of decision coaching, or positive experiences with decision coaching are important for participation and progress in decision making.

### CMO4: Three roles (i.e., decision coach, patient, clinician) share a common goal of the patient being involved in decision making

*When the three roles (i.e., decision coach, patient, clinician) are committed to patients' involvement in decision making (C), they will share a common goal (M), and as a result, work collaboratively as partners in decision making (O).* Five papers substantiated the theoretical statement in CMO4 [13, 36, 40, 45, 48]. In addition to healthcare providers’ commitment to patients’ involvement in decision making mentioned in CMO1, patients’ commitment to involvement is also crucial for the successful implementation of decision coaching. Davison and colleagues suggested that patients’ preferences for involvement in treatment decision making was not a static phenomenon [36]. When they have strong personal beliefs that external factors (such as the care from healthcare providers), rather than themselves, control their care or recovery pathways, they are less likely to be actively involved in decision making [48]. Overall, we propose that the commitment of the three roles (i.e., decision coach, patient, clinician) to patient involvement in decision making are likely to lead to their collaboration in addressing patients’ decisional needs.

### CMO5: Patients confide in the decision coach as a trusting relationship is built

*When there is a trusting relationship between a decision coach and a patient (C), the patient will confide in the decision coach (M), and as a result, progress in decision making (O).* Six papers underscored the importance of trust-building for effective decision coaching [13, 37, 42, 50, 51, 56]. Thom and colleagues considered trusting relationships as central to coaching support to patients—“ If I have a patient that doesn’t confide in me or that doesn’t trust me, I’m never going to get anything out of them”(page 512); “Trust makes patient more willing to confide in and accept support from health coach around health decisions” (page 513) [56]. The relationship between trust and coaching activities is iterative; trust makes coaching possible; coaching also builds trust [13, 56]. In a qualitative study conducted by Jull and colleagues on cultural adaptation of a shared decision making tool, they found that First Nations, Inuit, and Métis women wanted to “find a trusted person” rather than “a neutral person” for decision coaching [42]. In Berger-Höger and colleagues’ pilot study [13] and Rahn and colleagues’ trial [50], they both listed trust as one important outcome indicator.

### CMO6: The patient perceives their decisional needs are recognized by the decision coach

*When the decision coach uses a tailored approach to decision coaching, which starts with an assessment of patients’ decisional needs and tailors the coaching to address unmet needs, rather than using a standardized protocol-based approach (C), patients will perceive their needs are recognized (M), and as a result, progress in decision making (O).* Eleven studies explicitly described the use of a tailored approach for decision coaching and emphasized the flexibility of decision coaching process to address patients’ decisional needs [33, 36, 41, 42, 44–48, 53, 54]. For example, in a pre/post study conducted by Lawson and colleagues [44], two social workers provided tailored decision coaching sessions to child-parent dyads and triads on insulin delivery methods based on the child’s health literacy needs, and encouraged the child to speak first when working through the decision making process. The tailored approach, as Shepherd and colleagues suggested [53], can “enable a greater degree of patient‐centred care and support patients to be meaningfully involved in consultations” (page 708).

### CMO7: Patients acquire knowledge for making the decision

*When a decision coach discusses evidence-based information on options, benefits, and harms with a patient (C), the patient will have an opportunity to exchange information with the coach, ask questions, and acquire the knowledge they need to make an informed decision (M), and as a result, improve understanding of information, and the quality of the decision making process will be improved (O).* All synthesized papers included the discussion of evidence-based information with a patient as a component of decision coaching [11–13, 33–56]. To make informed decisions, patients need to gain a basic understanding of their health conditions, options, and the consequences of each option [56]. The delivery of evidence-based information could have different modes, ranging from patient decision aids [11–13, 43, 47], workbooks [33, 34], booklets [48, 49] to web-based information tools [50, 51]. Many of the included studies [11, 13, 34, 39, 43, 46, 47, 52, 54] confirmed that the discussion of evidence-based information increased patient knowledge.

### CMO8: The patient and decision coach reach a common understanding of patient’s values

*When a decision coach works with a patient to clarify the patient’s values for outcomes of options (C), the patient and decision coach will reach a common understanding of what matters most to the patient (M), and as a result, the patient becomes clearer on the values-based preferred option (O).* Twenty-four of 27 synthesized studies described the necessity of clarifying patient’s values during decision coaching [11–13, 33–37, 39–48, 50–52, 54–56] and three studies lacked explicit descriptions. Many included studies [33–35, 37, 43, 44, 46, 48, 55] referred to frameworks (e.g., ODSF, SCOPED) or tools (e.g., the Ottawa Family Decision Guide) to elicit patient values (including goals and priorities) within the decision coaching interventions. Eliciting patients’ values may contribute to achieving values-based decisions.

### CMO9: Patients feel supported when family and significant others participate in decision coaching

*When patients who prefer to work with supportive others, invite family members or significant others to participate in decision coaching together (C), they will feel supported (M), and as a result, select a preferred option (O).* Two papers contributed to the theoretical statement in CMO9 [36, 49]. When family members or significant others accompany the patient in decision coaching, the patient is more likely to feel supported and confident to make a decision [36]. However, it is also possible that family members’ values and preferences are inconsistent with the patient. This is problematic if they dominate the decision coaching conversations and negatively impact the patients’ free expression and decision outcomes. Therefore, we propose that decision coaches consider the potential contributions of family members to determine the need for sessions with and without them.

### CMO10: Patients process information and deliberate on options

*After a tailored decision coaching session(s), patients, especially those with complex decisional needs, may need time (C) to process information and deliberate on options (M). As a result, patients progress in decision making to reach a preferred option (O).* Six papers contributed to the theoretical statement in CMO11 [34, 38, 43, 48, 52, 53]. Shepherd and colleagues found that with repeated exposure to the decision coaching intervention, patients felt significantly better prepared for each consultation, reported higher decision self-efficacy and lower decisional conflict over time [53]. Three studies reported that over time (two weeks, 6 months, or 12 months after the decision coaching intervention), patients showed significantly higher decisional self-efficacy [38], lower decision regret [38], decreased decisional conflict [43, 52], and more patients had made the treatment decision [43]. However, McBride and colleagues revealed that for patients with a diabetic foot, the decision conflict increased over time (12 weeks) after receiving decision coaching [48]. As was explained by the authors, this population experiencing long-term conditions may have cultivated a negative belief that there is little they can personally control in their care and would become highly dependent on healthcare professionals in terms of healthcare decisions. The increased decision conflict observed over time may have been a result of participants engaging in a learning process from the decision coaching intervention with improved knowledge about different treatment options [48]. Overall, we propose that patients, especially those with complex decisional needs, often need time for deliberations on decision options to make progress in decision making.

### CMO11: Decision coaches share patients’ personal circumstances and advocate for their values for preferred options

*When a decision coach understands the impact of patient’s health condition on their quality of life and their values for outcomes of options (C), the decision coach will play a bridging role between the patient and clinician by sharing patient’s personal circumstances and advocating for patient’s values for preferred option (M), and as a result, improve patient-clinician communication (O).* Two papers contributed to the theoretical statement in CMO10 [53, 56]. With a thorough understanding of patient's health conditions and their values, a decision coach can act as the liaison between patient and clinician to facilitate communication and advocate for the patient [56]. The decision coach can strengthen the patient-clinician relationship and communication by “helping patient communicate with clinician”, “providing clinician with information about patient”, “clarifying clinician’s communication to patient”, “helping patient to disclose to clinician” and “reducing patient’s fear of physician” (page 513) etc. [56].

### CMO12: Decision coaches are supported by the leadership team with access to resources to fulfill their role

*When the organizational leadership team is committed to support patients' involvement in decision making (C), decision coaches will have access to resources (e.g., workload, training, patient decision aids) (M), and as a result, implement and optimize the process of decision coaching (O).* Nine studies contributed to the theoretical statement in CMO12 [11, 34, 37, 40, 41, 44, 45, 50, 56]. These studies reported that organizational factors, such as time [11, 13, 34, 41], resources [11, 40, 41], and staff shortage [41, 56] are crucial barriers to embedding decision coaching into current service procedures. Leadership support is proposed as a promising strategy to facilitate organizational learning and build a shared vision about the decision coaching role [45], therefore promoting implementation and optimization of decision coaching.

### Refined program theory proposition

**Fig. 3:**
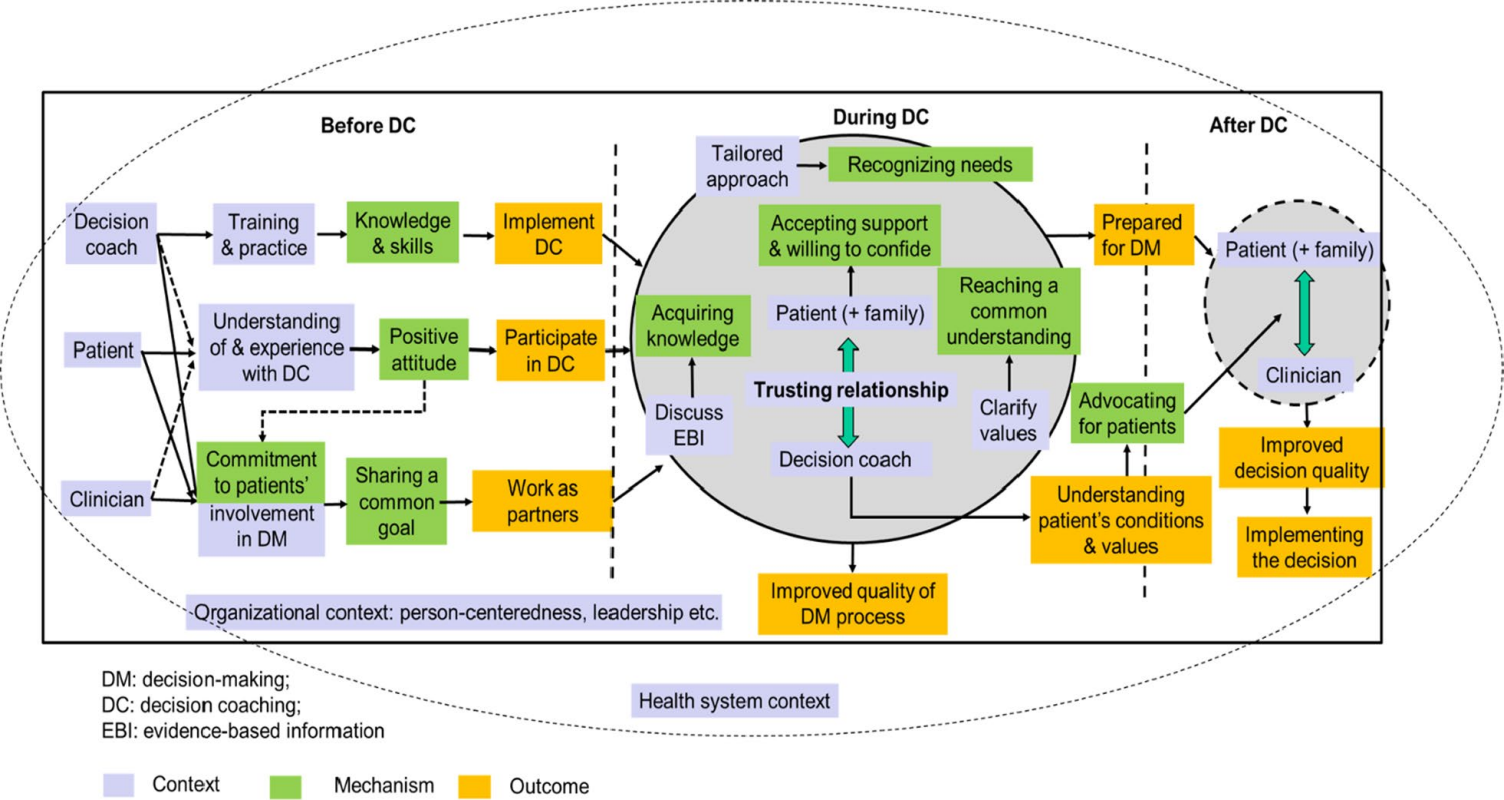
Refined program theory of decision coaching

Based on the 12 CMOs and the expertise of stakeholder group, we refined the program theory on how decision coaching supports patients’ progress in decision making (see Fig. 3). In the refined program theory, we separate decision coaching into three interconnected phases (i.e., before, during, after decision coaching) involving three key roles (i.e., decision coach, patient, clinician), which are all framed within the organizational setting and the broader health system context. For decision coaching to be initiated, decision coaches should have essential knowledge and skills; decision coaches, clinicians, and patients should be committed to patients' involvement in decision making (CMO1–CMO4). During decision coaching, the quality of decision making process and patients’ progress in decision making are related to the patient (i.e., willing to confide, perceiving their decisional needs are recognized, acquiring knowledge, feeling supported), and the patient-decision coach interaction (i.e., exchanging information, sharing a common understanding of patient’s values) (CMO5–CMO9). Patient progress in making or implementing a values-based preferred decision after decision coaching can be facilitated by the decision coach’s advocacy for the patient, and the patient’s deliberation upon options (CMO10–CMO11). At the organizational level, leadership support enables decision coaches to have access to essential resources to fulfill their role (CMO12). Patients may make a decision with or without a consultation with a clinician.

## Discussion

Our realist review of decision coaching synthesized 27 papers and generated 12 CMO configurations, which were used to refine the program theory of how decision coaching interventions activate patients’ progress in decision making. The 12 CMOs fit the before, during, after decision coaching stages, and the organizational context with three main roles involved—the decision coach, the patient, and the clinician. Key mechanisms for decision coaching to support patients’ decision making include (a) decision coaches’ knowledge and skills; (b) decision coaches’, patients’, and clinicians’ commitments to patients’ involvement in decision making; (c) patients’ willingness to confide, perception of their decisional needs are recognized, acquirement of knowledge, and feeling of being supported; (d) patient and decision coach’s information exchange and a common understanding of patient’s values; (e) decision coaches’ advocacy for patients; (f) patients’ deliberation upon options; and (g) decision coaches’ access to resources by leadership support. These findings lead to our discussions on three key points.

### Encouraging patients to participate in decision coaching

Decision coaching that supports patients to take an active part in decision making may be new and unfamiliar to patients. As our review suggests, patients need to be made aware of the availability of decision coaching and have the opportunity to ask questions about decision coaching, so that they are likely to value and engage with a decision coach. Our finding is consistent with the broad literature on shared decision making. The updated ODSF includes “invite (patient) participation” into the framework to highlight its importance in improving patients’ engagement with decision support [5]. In the Six-step Shared Decision Making Model proposed by Healthwise, “inviting the patient to participate” was listed as the first step to inform patients that they have options in decision making and their values and preferences are an important part of the decision [57, 58]. In a systematic review of patient-reported barriers to shared decision making, patients’ perceived their abilities to participate in shared decision making depended on being given permission to participate [59]. Patients, who have longstanding experiences with a paternalistic decision making approach, often desire to be a “good” patient, rather than “annoy” clinicians by asking questions and trying to be more involved [60]. Inviting patients to participate in decision coaching can change their perceptions of their roles and improve their experience with healthcare decision making.

### Building a trusting relationship between the decision coach and the patient

Decision coaching is relational and requires the building of trust between the coach and the patient so that their interaction can be initiated or enhanced [61, 62]. As Thoms and colleagues suggested, a trusting relationship is central to the decision coach’s support for a patient in that it enables the patient to express ideas, ask questions, exchange information, and makes the decision coaching effective [56]. Trusting relationships are especially important for decision coaching with populations who may prioritize personal connections with their healthcare providers in addition to information or facts to make decisions [42, 61, 63, 64]. In a recently published realist review, Waldron and colleagues identified that mutual trust between patients and healthcare providers was one of the key mechanisms to promote patients’ engagement in shared decision making [65]. Different from other decision support tools, such as patient decision aids, decision coaching often requires a more intensive conversation between the patient and the decision coach who may be a clinical team member or situated independently from the clinical program. Building trusting relationships may be more challenging for decision coaching because the patient and decision coach may be new to each other, yet still need to exchange information on personal conditions and values in a limited amount of time. In their longitudinal qualitative study conducted by Dang and colleagues, they found that from the perspective of persons with HIV infection, healthcare providers could turn to strategies such as providing reassurance, avoiding judgmental language and behavior, encouraging patients to ask questions, and listening with humility to build trust and rapport early in the new doctor-patient relationship [66].

### Leadership support

Although there are no studies systematically reviewing the barriers to implementing decision coaching, organizational restraints (e.g., lack of time and resources, staff shortage) are continuously reported as the main obstacles [40, 41, 50]. In a scoping review of organizational characteristics that influence the implementation of shared decision making, Scholl and colleagues classified the influencing factors into six categories: culture, leadership, organizational priorities, teamwork, resources, and workflows [67]. In a qualitative study, leadership support and multidisciplinary teams were viewed as critical strategies for implementing shared decision making [68]. The recently released shared decision making guideline by the National Institute for Health and Care Excellence has also listed high-level leadership as the first recommendation in the guideline to promote its implementation at an organizational level [69]. We propose that these influencing factors and strategies also apply to decision coaching. Leadership plays an important role in increasing acceptance of decision coaching, creating a supportive environment, and optimizing related policies. Johnson and colleagues suggested that the most cost-effective strategy to implement and scale-up decision coaching is to incorporate client-centred coaching into routine in-service training and pre-service training curricula [41]. These strategies rely on organizational leaders’ valuing the decision coaching role and ensuring adequate resources for decision coaches.

### Implications for decision coaching practice and research

Based on the 12 CMOs and the refined program theory, we offer nine suggestions for healthcare organizations and decision coaches: (a) attain leadership support for patient involvement in decision making; (b) attend training and practice decision coaching; (c) encourage patients to participate in decision coaching to change their attitudes and improve their experience; (d) build a trusting relationship with the patient; (e) use a tailored approach for decision coaching based on patients’ decisional needs; (f) discuss evidence-based information with the patient and clarify values; (g) discuss inviting family members to participate in decision coaching; (h) understand the influence of patients’ health condition and their values and act as the liaison between patients and clinicians; (i) give patients time to process information and deliberate on options after decision coaching sessions.

It is unclear the extent to which decision coaching has added benefits compared to evidence-based information (such as patient decision aids) alone [2–4]. Yet decision coaching, compared with evidence-based information, often requires larger investments from the organization, decision coaches, and patients. Based on one model on decision support [70] and one on healthcare service provision [71], we hypothesize that decision coaching may be especially necessary for patients with complex decisional needs who often need more tailored decision support. Further research should be conducted to discern which patient groups benefit more from decision coaching support to inform the distribution of decision support resources by healthcare organizations. There is limited information on the costs associated with decision coaching from the Cochrane review [2] and our realist review. Only one study within the Cochrane review conducted an economic evaluation and showed decision coaching was more cost-effective compared to the provision of evidence-based information by a patient decision aid or usual care [72]. We suggest future research includes health economics indicators to compare the cost-effectiveness of decision coaching and the use of evidence-based information approaches. In addition, it may also be important to investigate how and under what circumstances family members can contribute to decision coaching, or more broadly, patients’ decision making.

Very few qualitative and process evaluation studies have been conducted to explore patients’ perspectives on decision coaching and explain decision coaching successes and failures, which limited our understanding of its working mechanisms. More qualitative and process evaluation studies are needed in future research to further refine the program theory.

### Strengths and limitations of this study

This realist review goes beyond considering the effectiveness of decision coaching interventions and delves into the mechanisms of how decision coaching works for people making healthcare decisions. It provides more evidence for researchers and decision coaches on developing decision coaching interventions, and for patients and patient groups who wish to advocate for its use. This realist review was conducted to complement a Cochrane systematic review; the combination of these forms of knowledge synthesis is, at this time, a novel approach. We also engaged with different types of stakeholders in our review process, which strengthened our understanding of the decision coaching mechanisms.

The inclusion of randomized controlled trials was both a strength and a limitation. It was a strength in providing evidence on decision coaching outcomes, but these studies without a process evaluation may have limited our understanding of how decision coaching works in a real-world clinical practice context and offered limited information on the mechanisms. To minimize this limitation, we also conducted additional searches to identify other relevant papers to test our program theory including qualitative, mixed-methods, and process evaluation studies. It should also be acknowledged that we did not prioritize papers for synthesis based on their methodological quality, as our main goal was to find information from papers with strong CMO contributions. However, we turned to the analytic strategy of adjudication (based on the methodological strength and weakness of studies) to help explain opposing outcomes, which could to some extent strengthen our understanding of decision coaching mechanisms.

## Conclusion

In our realist review, we generated 12 CMO configurations and a refined program theory to build a causal understanding of how decision coaching supports patients’ progress in decision making. We offerred nine suggestions for decision coaches and healthcare organizations. For decision coaching to work for people making healthcare decisions, our findings suggest that there should be strong leadership support and commitment from decision coaches, clinicians, and patients. Decision coaches should attend training and practice to improve coaching capabilities, and encourage patients’ participation. During decision coaching, a trusting relationship is the foundation for tailored decision support. The decision coach can act as a liaison between patients and clinicians to facilitate patients’ progress in making or implementing a values-based preferred decision. More empirical studies, especially qualitative and process evaluation studies, are needed to further refine the program theory.

## Supplementary Information

**Additional file 1: Appendix A:** Demographic of executive and stakeholder group members. **Appendix B:** Initial program theory. **Appendix C:** Relevance rating checklist. **Appendix D:** Data extraction form.

## Data Availability

All data generated or analyzed during this study are included in this published article and its supplementary information files.

## Abbreviations

CMO: Context-mechanism-outcome
ODSF: Ottawa Decision Support Framework
PRISMA: Preferred Reporting Items for Systematic Reviews and Meta-Analyses
SCOPED: Situation–Choices–Objectives–People–Evaluation–Decisions
