# Supplementary material for: Understanding how and under what circumstances decision coaching works for people making healthcare decisions: a realist review
Source: BMC Med Inform Decis Mak. 2022 Oct 8;22:265. doi: 10.1186/s12911-022-02007-0 (PMC9548102; doi:10.1186/s12911-022-02007-0)
Supplement: Supplementary file 1 — Additional file 1: Appendix A: Demographic of executive and stakeholder group members. Appendix B: Initial program theory. Appendix C: Relevance rating checklist. Appendix D: Data extraction form. [file 12911_2022_2007_MOESM1_ESM.docx]

Appendix A: Demographic of executive and stakeholder group members

| Variables | | Group members (N=11) | |
| --- | --- | --- | --- |
| Decade of birth | 1950's | 2 | 18% |
|  | 1960's | 3 | 27% |
|  | 1970's | 3 | 27% |
|  | 1980's | 2 | 18% |
|  | 1990’s | 1 | 9% |
| Sex at birth | Female | 10 | 91% |
|  | Male | 1 | 9% |
| Gender | Woman | 10 | 91% |
|  | Man | 1 | 9% |
|  | Trans, fluid, or non-binary | 0 | 0% |
| Country | Australia | 2 | 18% |
|  | Canada | 4 | 36% |
|  | Other (China, Denmark, Germany, Japan, Norway) | 5 | 45% |
| Current level of education | Masters | 2 | 18% |
|  | PhD | 5 | 45% |
|  | Post doctorate | 4 | 36% |
| Primary language | English | 6 | 55% |
|  | Other (Chinese, Danish, German, Japanese, Not specified) | 5 | 45% |
| Experience(s) with decision coaching | Beginning to learn about decision coaching | 2 | 18% |
|  | Received decision coaching as an intervention | 2 | 18% |
|  | Gave decision coaching to someone making a decision | 8 | 73% |
|  | Was trained to use decision coaching | 7 | 64% |
|  | Provided training in decision coaching to others | 7 | 64% |
|  | Developed decision coaching interventions | 8 | 73% |
|  | Conducted research to evaluate decision coaching | 9 | 82% |
|  | Developed and/or promoted health policy that supports decision coaching | 3 | 27% |
|  | Synthesized evidence on decision coaching | 8 | 73% |

Appendix B: Initial program theory


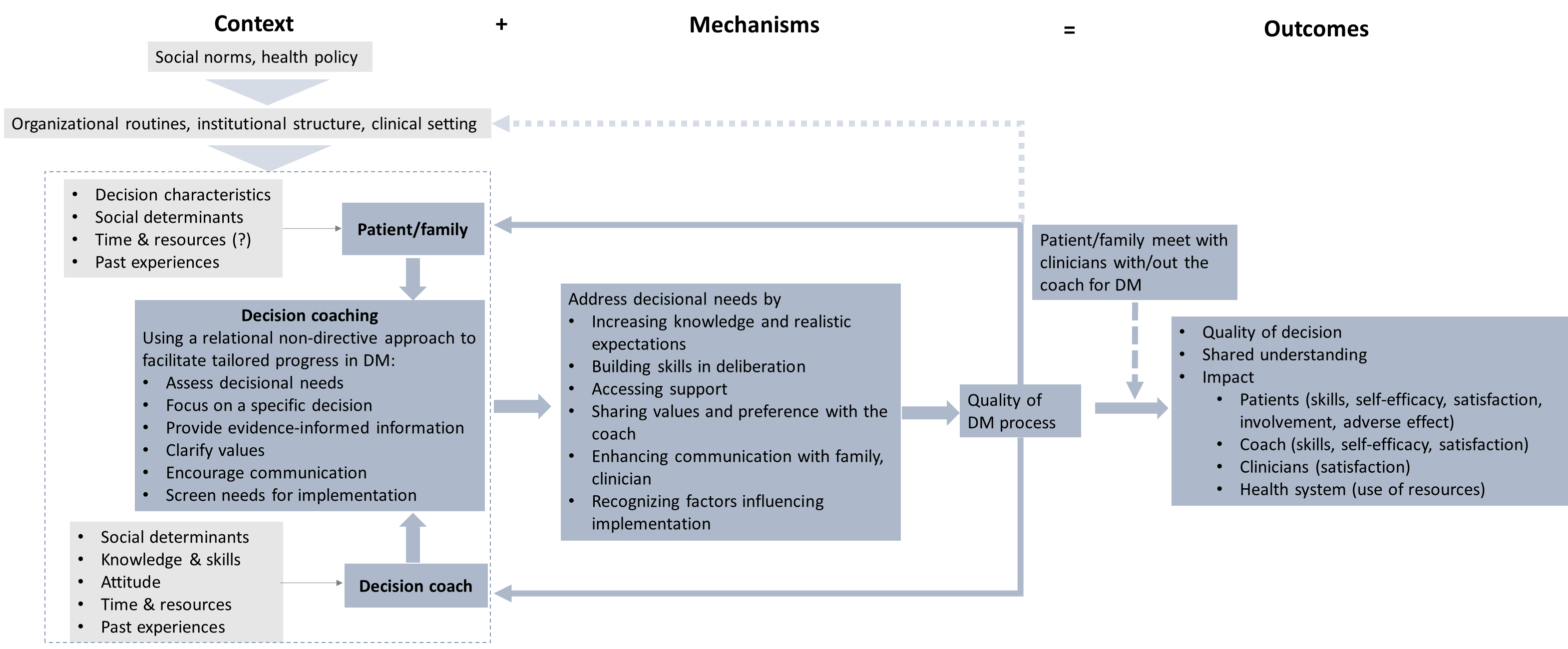


Appendix C: Relevance rating checklist

Level of relevance criteria

**Overall relevance (level 0-3) (level 3 precedes over level 2, level 1 and level 0)**

Level 3: the mechanism is rated level 3.

Level 2: the mechanism is rated level 2; or the context rated level 3.

Level 1: the mechanism is rated level 1; or the context rated level 2;

Level 0: the mechanism is rated level 0; or the context rated level 1 or 0; or cannot be excluded based on the eligibility criteria.

**Mechanisms (level 0-3)**

Level 3: the paper provides rich information on the mechanism: it directly answers the review question, i.e.: how/why/why not decision coaching works for people making healthcare decisions from patients’ or HCPs’ perspective; or is intervention studies on decision coaching with explicit theory (which has an explanatory focus) support; or investigates the experiences, perceptions of patients / people / HCPs / decision coaches on decision coaching; or investigates the barriers and/or facilitators to implementing decision coaching; or investigates the development/implementation process of decision coaching intervention.

Level 2: the paper provides some information on the mechanism: it investigates the reasons why decision coaching leads (or fails to lead) to certain outcomes (eg: improved quality of decision), or is intervention studies on decision coaching without explicit theory support.

Level 1: the paper provides a little information on the mechanism: it does not address the question on mechanism in the study, but only infers how decision coaching works in the discussion section.

Level 0: the paper provides no information on the mechanism: neither does the paper address the question on mechanism, nor does it discuss the mechanism.

**Context (level 0-3)**

Level 3: the paper provides rich information on the context based on the IPT: it investigates or explains the impact of different level contexts on decision coaching.

Level 2: the paper provides some information on the context based on the IPT: it investigates or explains the impact of certain level context (eg: individual context) on decision coaching.

Level 1: the paper provides a little information on the context based on the IPT: it only describes the decision coaching context in the method and/or results section.

Level 0: the paper provides no information on the context based on the IPT.

**Outcomes (level 0-3)**

Level 3: the paper reports different level outcomes based on the IPT.

Level 2: the paper reports certain level, or several outcomes based on the IPT.

Level 1: the paper focus on one specific outcome based on the IPT.

Level 0: the paper does not report any outcome based on the IPT.

Appendix D: Data extraction form

| **Basic characteristics** | | | | | | | | | | |
| --- | --- | --- | --- | --- | --- | --- | --- | --- | --- | --- |
| Author year | Country | Aim of study | conclusion | Study design |  |  |  |  |  |  |
| **Mechanism** | | | | | | | | | | **STOP?** |
| Guiding theory | How does theory inform the study | research rationale/hypothesis, perceptions/experience of Interventions, explanations for success/failure, Barriers/facilitators for implementation | Increasing knowledge and realistic expectations | Building skills in deliberation | Accessing support | Sharing values and preference with the coach | Enhancing communication with family, clinician | Recognizing factors influencing implementation | Other potential mechanisms | Is Mechanism well described? If no, then stop here |
| **Decision coaching intervention** | | | | | | | | | | |
| Setting | providers (+ # No.) | consumers (+ # No.) | Decision Type | Intervention components | Development process | how decision coaching is used | Patient meets with clinician? | Length of time to follow-up | Other information |  |
| **Context** | | | | | | | | | | |
| Individual-patient | Individual-decision coach | Organizational | System | Notes |  |  |  |  |  |  |
| **Outcomes** | | | | | | | | | | |
| Quality of DM process | | | | | | | | | | |
| Patient involvement in decision making | Decision conflict | other outcomes |  |  |  |  |  |  |  |  |
| Quality of decision | | | | | | | | | | |
| Preparation for decision making | Decision self‐efficacy | Decision regret | Stage of decision making | other outcomes |  |  |  |  |  |  |
| Impact-patient | | | | | | | | | | |
| knowledge | satisfaction with decision coaching | Satisfaction with care | other outcomes |  |  |  |  |  |  |  |
| Impact-coach | | | | | | | | | | |
| Impact-clinician | | | | | | | | | | |
| Health system: Length of consultation, Cost to health system | | | | | | | | | | |
| Your comments (optional): reflections on the potential relationships among intervention, mechanism, context, outcome | | | | | | | | | | |
